# Natural Products as Sources of New Analgesic Drugs

**DOI:** 10.1155/2022/9767292

**Published:** 2022-01-12

**Authors:** Arielle Cristina Arena, Candida Aparecida Leite Kassuya, Elisabete Castelon Konkiewitz, Edward Benjamin Ziff

**Affiliations:** ^1^Universidade Estadual Paulista, São Paulo, Brazil; ^2^Universidade Federal da Grande Dourados, Dourados, Brazil; ^3^New York University Grossman School of Medicine, New York, NY, USA

At the same time, as conventional therapies for the treatment of pain are losing their effectiveness, more and more pharmacological studies are showing that products of natural origin are promising for the development of new molecules or therapies. The idea for this special issue emerged from the considerable interest in identifying new therapeutic agents obtained from plants used in popular medicine for the treatment and/or management of pain. New drugs and therapies that are highly effective, low cost, safe, and available may be developed through comprehensive investigation of the bioactivities of a number of natural compounds. With this in mind, researchers from different countries were invited to contribute original research and review articles about new natural products with analgesic properties. After the review process, 5 high-quality articles were accepted for publication. The topics included in this issue comprise effects of new medicinal plants with analgesic properties studied using animal models of chronic pain, the safety and/or adverse effects of medicinal plants with analgesic activities, effects of new medicinal plants in pain management, and the mode of action of plants with analgesic activities. A brief summary of each of the accepted articles is provided as follows.

The paper by H. Ilmi et al. evaluated the analgesic and antipyretic effects of a tablet obtained from *Andrographis paniculata* ethyl acetate fraction, in animal models. *A. paniculata* is an herbaceous plant belonging to the Acanthaceae family, found especially in India, Sri Lanka, Pakistan, and Indonesia. The authors observed that the *A. paniculata* ethyl acetate fraction exhibited analgesic and antipyretic activities in the tablet dosage form. The results revealed that this species could be an excellent candidate as an herbal medicine for the treatment of pain and fever.

C. C. Liao et al. studied the effects of *Scutellaria baicalensis* (SB), a traditional Chinese medicine used for the treatment of inflammatory and painful conditions, on migraine by behavioral analysis of systemic administration to rats using the nitroglycerin (NTG) induced migraine rat model. The authors observed that pretreatment with 1.0 g/kg SB alleviated migraine-related behaviors in the NTG-induced experimental model. Thus, SB may be a promising natural product for the treatment of migraine.

In another paper, J. Chang et al. investigated the molecular mechanism as well as the effective compounds present in the Gu-tong formula. Gu-tong formula (GTF) is used in treatment of cancer-related pain and includes nine traditional Chinese medicines. The authors revealed the potential pharmacological mechanism of GTF in cancer pain treatment, from a systematic perspective, which may involve the secretion of inflammatory cytokines, membrane potential, bone protection, and other biological processes through the regulation of chemokines, MAPK, and TRP channels. Cholesterol and stigmasterol in GTF have been suggested to be the key pharmacodynamic molecules for analgesia, as seen in molecular docking screening. These findings provide insights into comprehending the synergistic effect of GTF on cancer pain relief.

The paper by S. A. Richard et al. focuses explicitly on an analgesic-antitumor peptide (AGAP) extracted from the venom of Scorpion BmK, with the aim of elucidating the cardinal analgesic and antitumor potentials of AGAP, with the emphasis on the key signaling mechanisms through which it functions. This review clearly points to the fact that AGAP has analgesic and antitumor potentials. Studies have shown that AGAP has a strong inhibitory influence on both viscera and soma pain. In addition, AGAP enhances the effects of MAPK inhibitors in neuropathic and inflammation-associated pain. In cancers such as colon cancer, breast cancer, lymphoma, and glioma, AGAP was effective in blocking proliferation. This molecule has more affinity for tumor cells and has less harmful effects on healthy cells. Thus, AGAP can represent a promising analgesic and antitumor therapy for some types of tumors.

In addition to therapeutic tests, products of natural origin should be evaluated for their safety, and toxicity tests are required by international regulatory agencies. W. A. S. S. Weerakoon et al. investigated the safety profile of the Sudarshana powder (SP) and its novel preparation, Sudarshana suspension (SS), in male Wistar rats, and tolerance studies were conducted with healthy adult volunteers. Sudarshana powder is an effective antipyretic Ayurvedic preparation, widely used in Sri Lanka as well as in India from the very early beginning of Ayurveda treatment. The data collected revealed that the extract of SP and novel preparation SS given orally to male Wistar rats and healthy volunteers did not cause toxicity at the therapeutic dose level, which demonstrates its safety after oral administration.

We hope that this issue of *Evidence-Based Complementary and Alternative Medicine* will be truly special for researchers studying the effects of natural products as sources of new analgesic drugs.

